# Intracellular energy production and distribution in hypoxia

**DOI:** 10.1016/j.jbc.2023.105103

**Published:** 2023-07-26

**Authors:** Darragh Flood, Eun Sang Lee, Cormac T. Taylor

**Affiliations:** Conway Institute of Biomolecular and Biomedical Research and School of Medicine, University College Dublin, Dublin, Ireland

**Keywords:** glycolysis, metabolism, hypoxia, ATP, bioenergetics, mitochondria, metabolon

## Abstract

The hydrolysis of ATP is the primary source of metabolic energy for eukaryotic cells. Under physiological conditions, cells generally produce more than sufficient levels of ATP to fuel the active biological processes necessary to maintain homeostasis. However, mechanisms underpinning the distribution of ATP to subcellular microenvironments with high local demand remain poorly understood. Intracellular distribution of ATP in normal physiological conditions has been proposed to rely on passive diffusion across concentration gradients generated by ATP producing systems such as the mitochondria and the glycolytic pathway. However, subcellular microenvironments can develop with ATP deficiency due to increases in local ATP consumption. Alternatively, ATP production can be reduced during bioenergetic stress during hypoxia. Mammalian cells therefore need to have the capacity to alter their metabolism and energy distribution strategies to compensate for local ATP deficits while also controlling ATP production. It is highly likely that satisfying the bioenergetic requirements of the cell involves the regulated distribution of ATP producing systems to areas of high ATP demand within the cell. Recently, the distribution (both spatially and temporally) of ATP-producing systems has become an area of intense investigation. Here, we review what is known (and unknown) about intracellular energy production and distribution and explore potential mechanisms through which this targeted distribution can be altered in hypoxia, with the aim of stimulating investigation in this important, yet poorly understood field of research.

The provision of sufficient biochemical energy is a vital requirement for any cell to carry out the active reactions necessary to maintain homeostasis. The energy needed for mammalian cell reactions is provided primarily through nucleoside triphosphate complexes. These complexes are comprised of a nitrogenous base combined with a ribose sugar to form the respective nucleoside structure. A phosphoester bond then allows binding of an inorganic phosphate group to the nucleoside, and further additional inorganic phosphate groups are bound to the preceding phosphate groups by phosphoric anhydride bonds, for example X-monophosphate, X-diphosphate, and X-triphosphate where X is the respective nucleoside structure. Energy is provided by these molecules through hydrolysis of the phosphoric anhydride bonds. With five naturally occurring nitrogenous bases, five separate natural nucleoside triphosphate complexes are possible. ATP is the main energy source of the cell, and it fuels most active processes (1, 2, 3) in addition to its role in intracellular signaling activities. These involve functions as diverse as activation of kinases (4) and signaling pathways (5) as well as cell-specific functions ranging from the activation of ion channels (6, 7) to the propulsion provided by flagella (8, 9). Hydrolysis of ATP typically yields a Gibbs free energy of −7.3 cal/mol (10) (Fig. 1).

**Figure 1:**
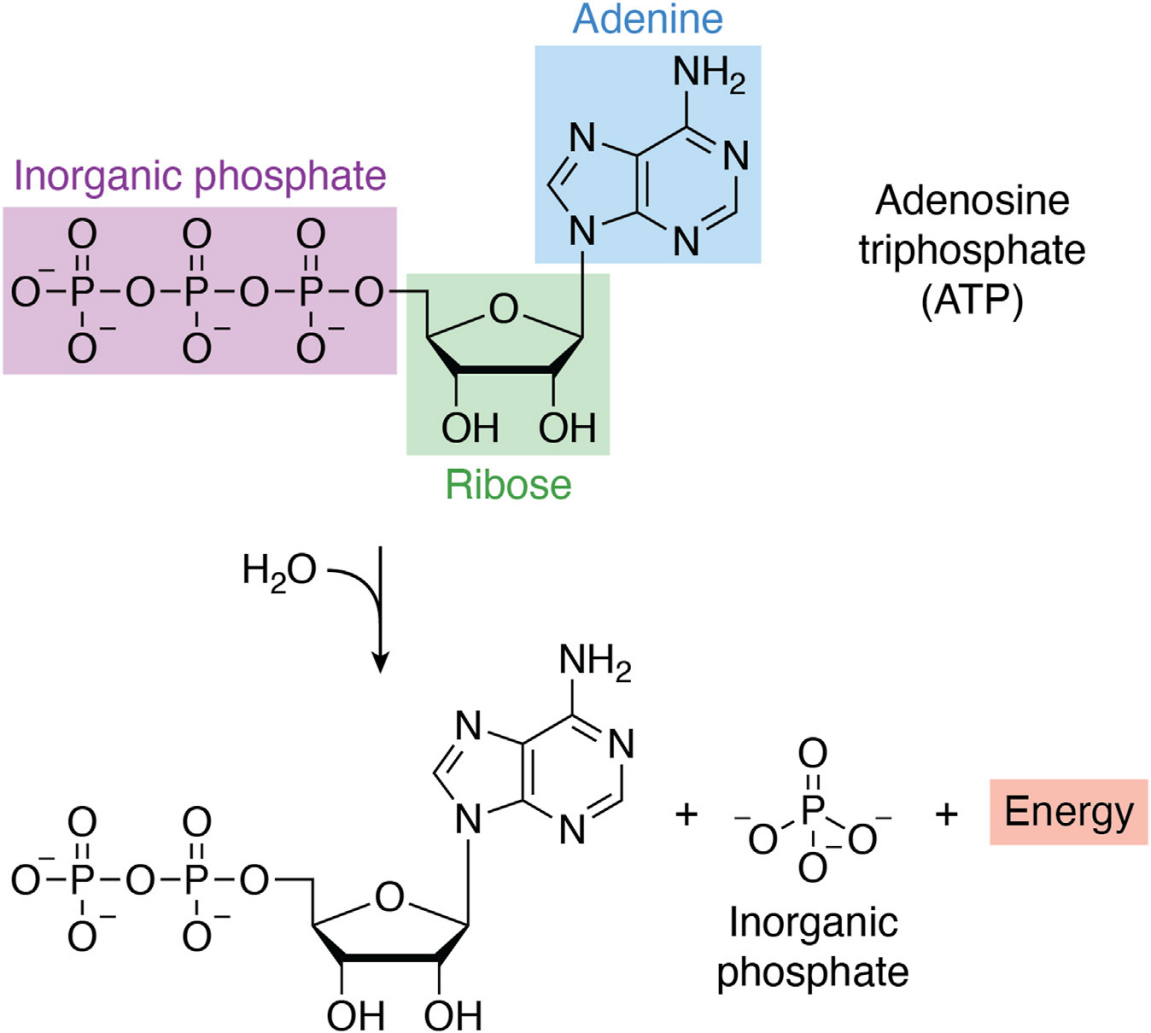
**Diagram showing the hydrolysis of adenosine triphosphate (ATP) into adenine diphosphate (ADP), energy, and inorganic phosphate.** Upon hydrolysis of ATP with H_2_O, the phosphoric anhydride bond is broken and releases energy causing the formation of ADP and an inorganic phosphate molecule.

GTP is involved in iron homeostasis (11), assembly of microtubules (12), involvement in GTP-binding signal transducing proteins (13), and energy homeostasis in some cancers (14). Cytidine, thymidine, and uridine triphosphate (CTP, TTP, and UTP, respectively) also have important roles (15, 16, 17), namely the formation of DNA and RNA synthesis through their deoxynucleotide form. While each nucleoside triphosphate has important functions in the mammalian cell, it is unclear how and why nucleosides have developed to play distinct roles during evolution with respect to cellular bioenergetics and metabolism.

However, the reason as to why ATP arose to become the universal energy currency in cells remains unknown. It is hypothesized that prebiotic synthesis of ATP is chemically favourable in aqueous solution in prebiotic conditions and thus led to the universal conservation of this molecule as a source of energy (18). Alternatively, a recent review paper has expertly discussed the relevant molecules needed to synthesise ATP and have postulated that the prebiotic synthesis of ATP could have been volcanism-dependent and became a direct energy source as enzyme-catalysed reactions replace abiotic ones (19). Yet, while acknowledging other nucleoside triphosphates and their contribution to normal physiological function, ATP is utilised as the main energy source of mammalian cells and will be focus of this review.

## ATP synthesis

The synthesis and production of ATP occurs mainly through the oxidative metabolism of glucose *via* respiration. This well-described model of respiration is highly efficient in yielding ATP with 38 molecules being produced per glucose molecule consumed (20, 21, 22, 23, 24, 25). Respiration is the principal mechanism of ATP generation in mammalian cells. This process first involves the transport of glucose into the cell *via* glucose transporters (GLUTs). There have been 14 identified GLUTs all of which play a role in glucose transport between compartments in mammalian cells (26). Upon transport of glucose into the cell, respiration begins with glycolysis and yields a net gain of two ATP molecules per molecule of glucose consumed. Alongside this ATP production, glycolysis also produces two molecules of pyruvate which can be utilized in the tricarboxylic acid (TCA) cycle and electron transport chain (ETC). The TCA cycle and ETC are located within the mitochondria and yield a further 36 molecules of ATP, provided that there is sufficient oxygen available as the final electron acceptors of the ETC.

Ketone body metabolism and beta oxidation are also important pathways which result in the production of ATP, as both pathways produce acetyl-coA which then feeds into the TCA cycle within the mitochondria (27, 28). While ketone body metabolism and beta oxidation are recognized mechanisms of ATP production, they will not be further explored in the context of this review. Instead, the focus will be on mitochondria and the glycolytic pathway as the main sources of cellular ATP.

Research regarding ATP has been heavily revolved around the synthesis, functions, and processes that ATP is involved in. However, there remain major questions concerning whether and how ATP is distributed inside the cell to fuel active cellular processes efficiently and effectively in subcellular microenvironments.

## The “ideal” state of intracellular energy distribution

The distribution/trafficking of intracellular ATP remains incompletely understood and with a lack of published studies concerning this topic, this review aims to highlight what is known thus far regarding intracellular energy production and distribution and then propose potential mechanisms by which directed transport can occur.

**Figure 2:**
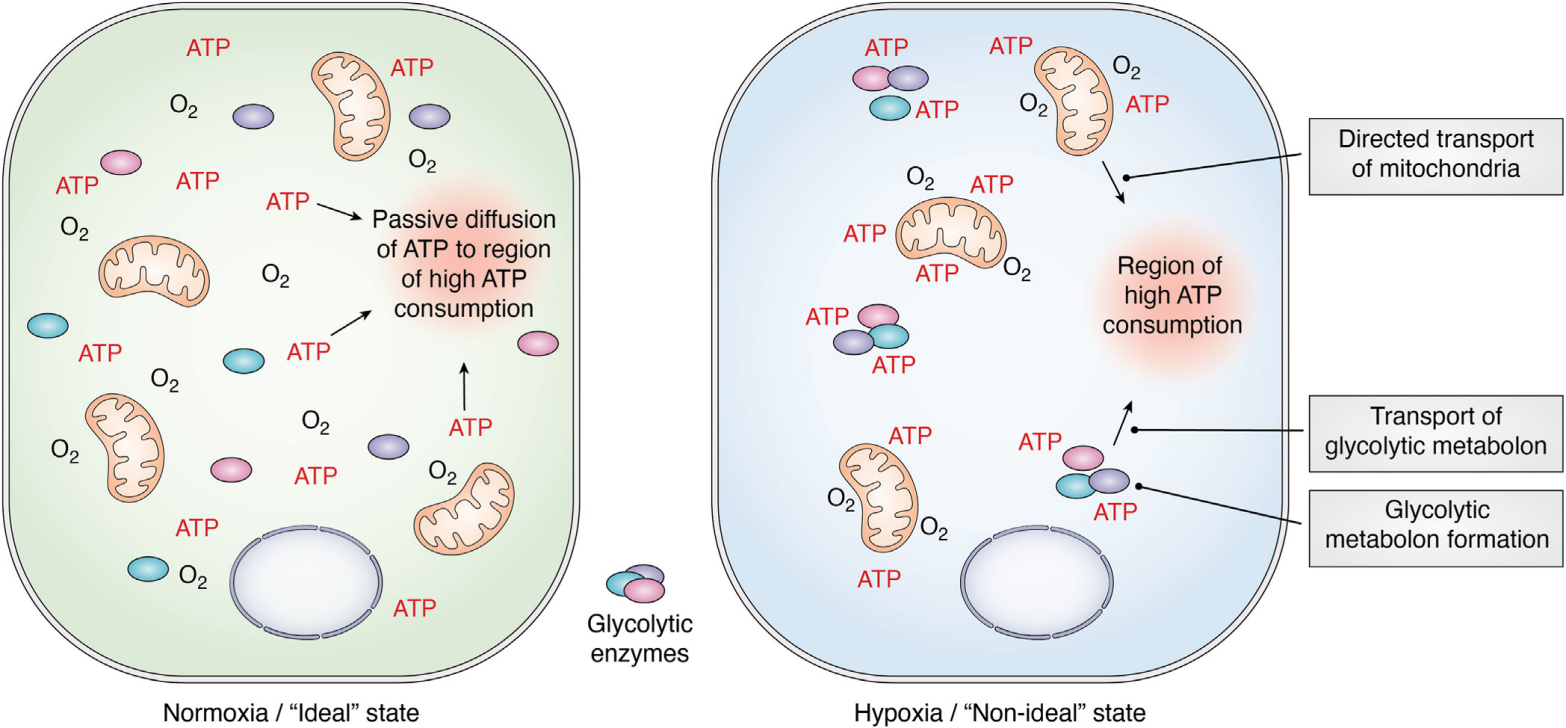
**Schematic demonstrating the ATP concentrations in the ideal state *versus* the hypoxia-induced nonideal state in a cell.** Mitochondria and glycolytic enzymes can be seen in both states as the ATP-producing systems which create the homogenous ATP concentration seen in the ideal state and the localization of regions of high ATP concentrations in the hypoxia-induced nonideal state. Decreases in ATP concentration due to hydrolysis are quickly restored *via* passive diffusion of ATP from regions of high concentration to low in the ideal state. The hypoxia-induced nonideal state section of the schematic demonstrates a hypothetical situation where decreases in ATP production or increases in consumption can result in regions of low ATP concentration. ATP is then localized to sites of production such as the mitochondria and glycolytic enzymes. Decreased ATP production results in a decreased intracellular concentration and so compromises the ideal state of passive diffusion. Regions of insufficient ATP concentrations are created which requires targeted intracellular energy trafficking. These regions of low intracellular ATP could be equilibrated *via* the formation and transport of a glycolytic metabolon or *via* the redistribution of mitochondria in the cytoplasm.

With the physiological intracellular concentration of ATP being approximately 4.4 mM (29), one theory is that this concentration exceeds the amount needed at local sites for normal cellular processes. According to this “idealized model” or “ideal state”, which is the simplest manner in which a dynamic system can be represented (30), the distribution of ATP occurs through passive diffusion across intracellular cytoplasmic space. In this model, as ATP is consumed in subcellular microenvironments, the concentration in that area is depleted but soon restored through passive diffusion of ATP from subcellular regions of high ATP concentration. The “ideal state” allows cells to continue all ATP-dependent tasks with any rise in bioenergetic demand being met with an intracellular ATP increase to compensate. This allows the passive distribution of ATP to continue and maintain energy homeostasis across the cell, as ATP concentration remains in excess of cellular demand. It is possible that this may be an effective process for cells in physiological conditions. However, as cells undergo hypoxia, ATP production is reduced due to reduced mitochondrial metabolism. Hypoxia then presents a “non-ideal state” in which passive distribution of ATP is insufficient to provide subcellular components with the energy required for their respective processes. While the “ideal state” *versus* the “nonideal state” paradigm is used to understand the basic concept of cellular energy distribution in this review, it is likely more complex as it is a dynamic system which fluctuates physically, spatially, and temporally between the “ideal” and “nonideal” states (Fig. 2).

The hypothesis of distinct intracellular microenvironments in the ideal and nonideal state is another complex phenomenon to consider. This can arise due to the diffusion restriction that organelles and other cellular structures impose on enzymes and molecules such as ATP. As the creation of ATP-depleted microenvironments may occur under normal physiological conditions, it is possible (indeed highly likely) that cells possess mechanisms that allow directed distribution of ATP-producing systems to regions of high ATP requirement to increase the availability and utilization of energy.

Mathematical studies have modeled the dynamics of enzyme-enzyme interactions, enzyme-substrate interactions, and substrate channeling (30, 31, 32, 33, 34, 35) in which most agree upon the principle that intracellular distribution of these interactions, intermediates, and products cannot rely solely on passive diffusion. It is therefore likely that this concept applies to the distribution of ATP-producing systems to sites of high energy demand. This could include the possible formation of metabolic complexes involving the glycolytic enzymes and/or movement of mitochondria. Yet, there remains a deficit in our understanding of intracellular energy distribution. Forms of cellular stress which inhibit the respiratory capabilities of the cell and reduce the production of ATP, provide a model for investigations into these proposed interactions and systems relating to energy distribution. Metabolic stress can be induced by hypoxia and can bring upon a marked reduction in ATP production *via* inhibition of mitochondrial respiration. In mammalian cells, hypoxia causes a decrease in ATP concentration by up to 30% (36). This review will focus on the cellular response to hypoxia and how the cell may adapt to overcome this nonideal state of ATP distribution. Currently there is a lack of evidence to support the targeted distribution of intracellular energy. Despite this, by understanding hypoxia and the concept of the “nonideal state” of energy distribution, potential mechanisms may be identified for further investigation. We believe this is an area of fundamental biologic importance which remains poorly understood and is in need of further investigation.

## Cellular response to hypoxia

As described above, hypoxia occurs when oxygen demand exceeds supply. Due to the importance of molecular oxygen in respiration, adaptation to hypoxia is vital for metazoan cell survival. A primary mechanism underpinning adaptation to hypoxia are transcription factors which induce adaptive gene expression. Primary among these hypoxia-induced transcription factors is the hypoxia-inducible factor (HIF) (37).

HIF is a heterodimeric protein which is comprised of a labile, oxygen-sensitive α-subunit, and a stably expressed β-subunit otherwise known as the aryl hydrocarbon receptor nuclear translocator or HIF-1β. There are three HIF-α isoforms (HIF-1α, -2α, and -3α) which have been described (38, 39, 40). This review will focus on HIF-1α which is widely expressed, whereas HIF-2α has been shown to be more cell-type specific with restricted tissue expression patterns (41).

In the presence of oxygen, Fe^2+^, 2-oxoglutarate, and ascorbate (42, 43, 44), prolyl hydroxylases (PHD1, PHD2, and PHD3) cause the degradation of HIF-1α by hydroxylation at two proline residues (Pro402 and Pro564) located within the oxygen-dependent degradation domain of HIF-1α (42, 45, 46). The hydroxylation of these residues promotes binding to von Hippel Lindau protein which is a component of the E3 ubiquitin ligase complex that tags the HIF-1α-subunit for proteasomal degradation (47, 48, 49, 50). Factor inhibiting HIF is another hydroxylase enzyme which hydroxylates an asparaginyl residue (Asn803) which is in the C-terminal transactivation domain of HIF-1α (51, 52). This prevents the interaction of the C-terminal transactivation domain with other transcriptional cofactors while in the presence of oxygen, 2-oxoglutarate, Fe^2+^, and ascorbate (53). As a result of these oxygen-dependent modifications, HIF-1α is degraded *via* the ubiquitin-dependent proteasomal degradation pathway (54, 55, 56, 57, 58, 59).

**Figure 3:**
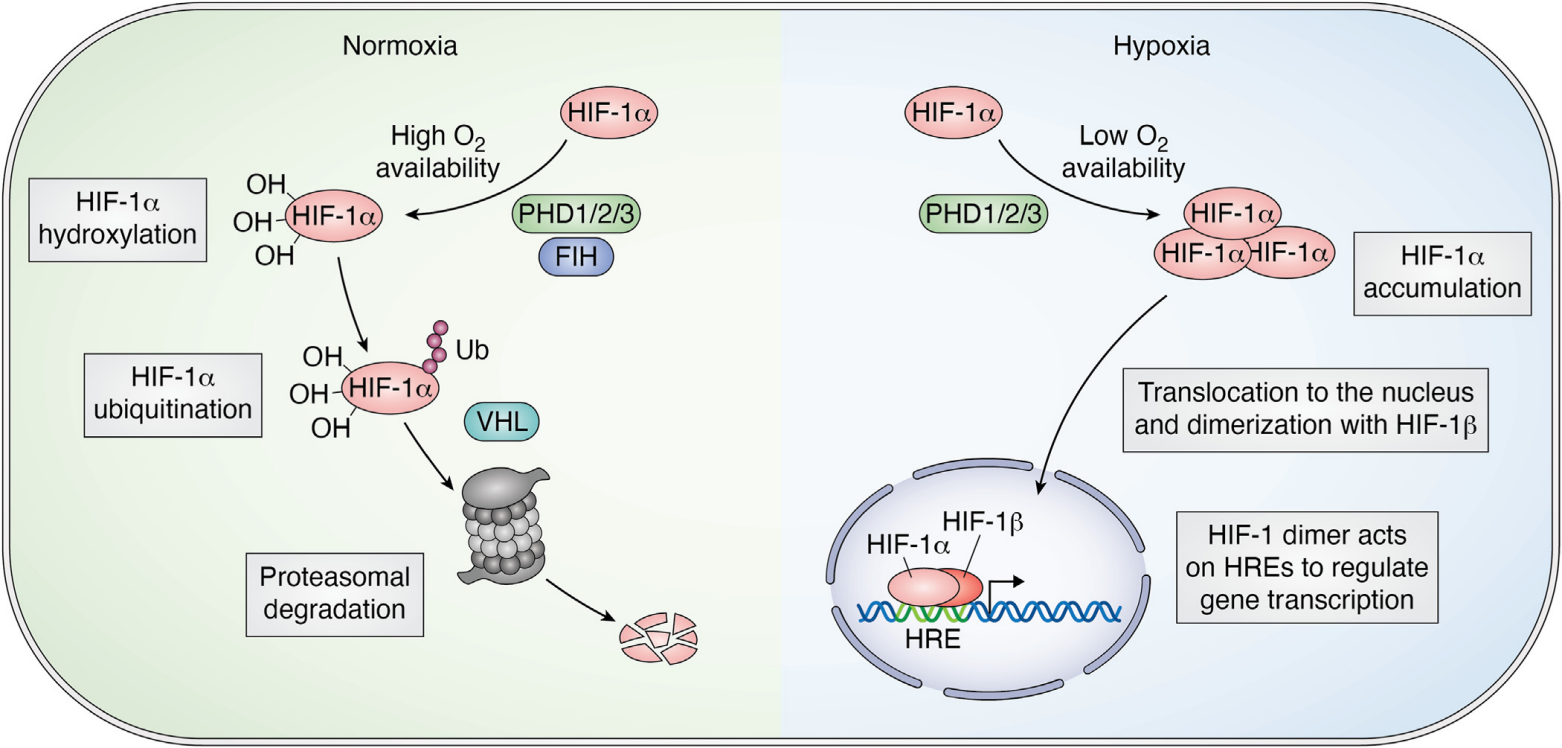
**Diagram showing the degradation and stabilization of the HIF-1α protein in the presence or absence of oxygen, respectively.** HIF-1α is degraded in the presence of oxygen *via* ubiquitin-dependent proteasomal degradation pathway upon hydroxylation, through FIH and PHDs, and ubiquitination *via* VHL. In hypoxic conditions, lack of oxygen prevents proteolytic degradation and dimerization of HIF-1α with HIF-1β/ARNT then allows for binding to hypoxia response elements on DNA and elicits a transcriptional effect to upregulate the production of the protein as a response to hypoxia. ARNT, aryl hydrocarbon receptor nuclear translocator; FIH, factor inhibiting HIF; HIF, hypoxia-inducible factor; PHD, prolyl hydroxylases; VHL, von Hippel Lindau.

In hypoxia, the oxygen-dependent hydroxylation of HIF-1α cannot occur, and this promotes the stabilization of HIF-1α and its accumulation in the cytoplasm. Upon stabilization and accumulation, HIF-1α is then translocated to the nucleus of the cell where dimerization with HIF-1β/aryl hydrocarbon receptor nuclear translocator occurs (60). Once the dimer is formed, HIF-1 then binds to hypoxia response elements located in promoter or enhancer regions of >500 genes including vascular endothelial growth factor (61) and erythropoietin (62). These genes are then transcribed to facilitate hypoxic adaptation (60, 63) (Fig. 3).

Further information regarding the HIF pathway and its implications in health and disease has been reviewed elsewhere recently (64). While HIF is the primary transcription factor that mediates responses to hypoxia, there are other responses which are also mediated by hypoxia. Other transcription factors which are sensitive to hypoxia include nuclear factor kappa-B (65, 66, 67, 68, 69), cyclic AMP response binding element (70, 71), activating protein 1 (72, 73, 74), and others which have been reviewed in detail elsewhere (75, 76, 77).

Other oxygen-sensitive pathways which are independent of transcription factors have also been described. AMP-activated protein kinase (AMPK) signaling is a primary oxygen sensor in mammalian cells. AMPK provides cells with a mechanism to adapt to low energy situations signaled by an increase in AMP to ATP ratio. Increases in the AMP:ATP ratio activates AMPK and allows for adaptation by decreasing energy expensive anabolic processes and increasing catabolic reactions (78, 79, 80). There is also an interconnected relationship between AMPK and HIF which has been reviewed in detail elsewhere (81). Other pathways include the suppression of energy-expensive protein synthesis *via* protein kinase R-like endoplasmic reticulum kinase (82) and mechanistic target of rapamycin complex 1 (83). While these pathways are important in overall energy metabolism, they will not be covered in further detail in this review.

## Hypoxic adaptations in the “nonideal state”

Hypoxia presents the cell with a unique challenge in that it does not allow the maintenance of the “ideal state” model of energy distribution reliant on passive diffusion of ATP due to diminished mitochondrial ATP production and results in the development of the proposed “nonideal state” model of intracellular bioenergetics. Induction of the “nonideal” state can allow for exposure of the mechanisms by which intracellular energy is distributed more efficiently through the formation or movement of ATP-producing systems to subcellular microenvironments of high ATP consumption. These systems which yield energy in the form of ATP can be influenced directly or indirectly by transcription factors such as HIF-1 and by other nontranscriptionally mediated adaptations to hypoxia. During hypoxia, important HIF-dependent adaptations are those of ATP-producing systems including the mitochondria and the glycolytic pathway.

### Mitochondrion—the powerhouse

The mitochondrion is the “powerhouse” of the cell producing 36 molecules of ATP per molecule of glucose metabolised. In hypoxia, mitochondrial ATP production is reduced due to the lack of available oxygen and as a result aerobic ATP is decreased (36). Additionally, further hypoxic adaptions which decrease the activity of the mitochondria are present in the cell including the HIF-1 dependent upregulation of pyruvate dehydrogenase kinase 1. Pyruvate dehydrogenase kinase 1 promotes the phosphorylation of pyruvate dehydrogenase, mediating the inhibition of pyruvate as a substrate for the TCA cycle, causing a reduction in the consumption of oxygen by the mitochondria (84, 85). In addition, HIF-1 inhibits mitochondrial biogenesis through the suppression of c-Myc leading to decreases in PCG-1β expression which is responsible for a loss in mitochondrial mass (86). While reductions in mitochondrial biogenesis and oxygen consumption can be seen as a protective mechanism against oxidative stress by reducing the production of reactive oxygen species (87, 88), it likely challenges the cell to overcome an intracellular energy deficit due to the reduction in activity of the mitochondria.

Although hypoxic adaptations of the mitochondria exist primarily to reduce oxidative stress (but resulting in decreased ATP production) a lower level of oxygen-dependent respiration could remain in the cell depending on the degree of hypoxia. Considering this, additional adaptations increase the output of mitochondrial ATP production despite the low oxygen availability. One such adaptation is the HIF-1-dependent enhancement of mitochondrial respiration efficiency *via* changes to the cytochrome oxidase subunits from the predominant normoxic COX4-1 isoform, to the hypoxia-induced COX4-2 isoform which improves the efficiency of respiration (89). Alongside this increase in COX4-2 expression, HIF-1 also promotes the degradation of COX4-1 through LON protease (90) allowing for the efficient COX4-2 isoform to become predominant throughout hypoxic periods.

With limited ATP production by the mitochondria, ATP distribution and utilization quickly becomes a vital factor in maintaining bioenergetic homeostasis. Directed localization of the mitochondria to regions of high ATP demand within the cell could improve the usage and production rather than relying on passive diffusion to these regions in need of energy. Spatial reorganization of mitochondria has been demonstrated to involve the HIF-1-dependent upregulation of a mitochondrial protein termed hypoxia-upregulated mitochondrial movement regulator (HUMMR). HUMMR is localized to the mitochondria of astrocytes to influence the anterograde transport of mitochondria, and upon loss of HUMMR or HIF-1 function, a significant decrease in mitochondrial number was observed in axons which were exposed to hypoxia (91). Furthermore, studies investigating the antiapoptotic protein survivin, demonstrated that survivin is increased by hypoxia (92, 93, 94), through HIF-1α (95). Survivin also mediates subcellular mitochondrial trafficking to the cortical cytoskeleton in periods of hypoxia in prostate cancer PC3 cells (96). The reorganization of the mitochondria to the cortical cytoskeleton near focal adhesion complexes was proposed to aid in fueling the energy-intensive movements of tumor cells and regulate tumor cell invasion and metastasis (96).

In combination with the altered localization of mitochondria, mitochondrial network morphology must also be considered. As it is known that the mitochondria can exist as highly branched tubular networks (97, 98) the effects of this on energy production and distribution is the key. These mitochondrial networks can differ in morphology and are dependent on tissue-type, cell-type, and the energetic needs of the cell and can exist in heterogeneous states (97, 99, 100, 101, 102, 103). In hypoxia, the mitochondria can appear donut-shaped, shortened, demonstrate perinuclear redistribution (104) and mitochondrial fission mediated by a mitochondrial protein Fun14-domain protein 1 (105). However, the effects and implications of the mitochondrial network or mitochondrial autophagy on the distribution of intracellular ATP to intracellular microenvironments of high demand are not yet fully understood.

Overall, in response to hypoxia, the mitochondrial adaptations mediated by HIF-1 primarily decrease activity of these organelles to prevent the production of reactive oxygen species. Along with this, other adaptive responses can increase the efficiency of respiration during periods of hypoxia and to spatially rearrange mitochondria which may optimize the mitochondrial network as well as the distribution of ATP to sites of high demand. In summary, hypoxia leads to a reduced but more efficient and spatially directed mitochondrial ATP production.

### Glycolysis

Due to net mitochondrial ATP production in hypoxia being reduced, the cell turns toward other energy-producing systems such as glycolysis to compensate for decrease in the intracellular levels of ATP.

These glycolytic hypoxic adaptations include the HIF-directed transcriptional upregulation of ATP-producing and rate-limiting glycolytic enzymes including phosphoglycerate kinase, aldolase, lactate dehydrogenase-A, enolase, and phosphofructokinase live-type as well as GLUT1 and 3 (106, 107, 108, 109, 110). The increased HIF-1α-dependent transcription of glycolytic enzymes and GLUT drive increased the uptake of glucose and activity of the glycolytic pathway during periods of hypoxia. While this upregulation of glycolytic enzymes increases the production of ATP, glycolysis may still be considered as an inefficient method of ATP production due to the relatively low ATP yield and the intermediate substrates produced relying on passive diffusion in the “ideal state” of energy distribution to allow for pathway activity. Directed channeling of glycolytic intermediates to the correct enzymes may provide a solution to increase the efficiency of glycolysis in terms of ATP production.

Along with previously described mathematical studies demonstrating models of enzyme-enzyme interactions, enzyme-substrate, and substrate channeling (30, 31, 32, 33, 34, 35), data have been presented in several studies that supports the hypothesis that substrate channeling of intermediate products involving glycolytic enzymes exists in various other species such as *Arabidopsis* (111, 112), *Protista* (113), *Saccharomyces cerevisiae* (114), and *Drosophila* (115). More recently, evidence supporting the formation of a glycolytic “metabolon” in mammalian cells such as human cervix adenocarcinoma cells and breast carcinoma cells (116), cardiomyocytes (117) and erythrocytes (118) has been proposed. Furthermore, hypoxia-dependent small ubiquitin related modifier-1 (SUMO-1) posttranslational modifications cause the colocalization of glycolytic enzymes, GAPDH and pyruvate kinase, to facilitate substrate channeling to increase the efficiency of glycolysis (119). Studies carried out on *S. cerevisiae* and human hepatocarcinoma cells have shown a direct link between “metabolon” formation and hypoxic stress in that glycolytic enzymes colocalize under hypoxic stress conditions and form glycolytic bodies or G-bodies (120, 121). Alongside this, a HIF-1-dependent metabolic complex has been recently identified in colonic epithelial cells to increase glycolytic metabolism in the absence of functional transcriptional machinery (122), further adding to the direct link between hypoxic stress and glycolytic “metabolon” formation. Substrate channeling and colocalization of these glycolytic enzymes present a potential mechanism whereby spatial and temporal reorganization of this glycolytic “metabolon” may serve to facilitate the distribution of intracellular energy in hypoxic mammalian cells. Yet, the mechanism by which this colocalization (hypoxia-dependent or hypoxia-independent) occurs remains unclear.

In recent years, many studies have focused on the idea of liquid-liquid phase separated (LLPS) biomolecular condensates which could provide a structured basis for how the glycolytic substrates and enzymes are colocalized. These condensates compartmentalize proteins and nucleic acids into membrane-less bodies with specific functions (123). With the ability to dissociate and reform within the cytoplasm of the cell, this allows the hypothetical “metabolon” to form spatially and temporally where it needs to be within the cell. Further analysis on the regulation of glycolysis *via* hypoxia and HIF-1α can be found here (124).

## Potential mechanisms of transport and spatial reorganization

While studies have identified the interaction of HUMMR and survivin with mitochondria and movement of these organelles (91, 96) alongside the colocalization of glycolytic enzymes in various species (111, 112, 113, 114, 115) and mammalian cell types (116, 117, 118), further investigation into the mechanisms of transport and spatial reorganization of these ATP-producing systems is warranted. It is important to note that these mechanisms may be mediated *via* adaptations to hypoxia much like the mitochondria and glycolytic enzymes have seen above, due to the reduced availability of intracellular ATP. Cytoskeletal rearrangement could eliminate diffusion restricted enzymes and allow for the transport or relocation of ATP-producing systems. Previously mentioned posttranslational modifications such as SUMOylation could influence the colocalization of glycolytic enzymes or affect other pathways which may prove bioenergetically favorable. Intracellular transport that occurs *via* chaperone proteins is another mechanism which the cell could utilize to allow for efficient ATP homeostasis by facilitating transport of ATP-producing systems both spatially and temporally. Biomolecular condensates or membrane-less organelles that form *via* LLPS have been an intense field of study in the last number of years and could serve as another mechanism in the formation and trafficking of ATP-producing system to sites of high intracellular energetic demand. These potential mechanisms are discussed in further detail below.

### Cytoskeleton

The potential role of the cytoskeleton in directing subcellular energy/ATP distribution is poorly understood. The cytoskeleton of mammalian cells provides mechanical support, an intracellular transport system and structure to facilitate cell division and movement. Most important are the reorganization capabilities of the cytoskeleton and transport capabilities the microtubules provide to distribute organelles and other subcellular components. In response to hypoxia, the filamentous actin component of the cytoskeleton reorganizes from a web-like structure into a parallel formation of stress fibers, while also increasing in size and number (125). This long-term reorganization of filamentous actin is also seen when cells are treated with dimethyloxallyl glycine, a PHD-inhibitor, indicating that the reorganization and formation of stress fibers is HIF-1α-dependent (126). Potentially, the reorganization of these fibers could alter the distribution of subcellular organelles, enzymes, and proteins within a cell and localize substrates and enzymes as a form of substrate channeling.

Studies into the microtubules have produced conflicting results. While some studies propose that hypoxia results in microtubule stabilization (127, 128) and other studies report that hypoxia results in the depolymerization of microtubules (129, 130, 131). This response of the microtubules could be due to cell-type specificity, degree, and duration of hypoxia or various other complex reactions which occur within the microtubular networks of these cells. Microtubule partitioning is another concept which has been reviewed and indicates that there may be changes in bioenergetics with a shift toward either soluble or polymerized microtubule pools (132). This partitioning hypothesis may allow for substrate channeling or directed transport of ATP producing systems. Though this hypothesis has yet to be fully tested.

The third component of the cytoskeleton is the intermediate filaments which consist of many proteins including vimentin, keratins, desmin, and neurofilaments and glial fibrillary acidic protein. Though intermediate filaments are primarily recognized for their role in withstanding mechanical stress and integrating components of the cytoskeleton, they also influence the organization of the internal structure of the cell (133, 134). Hypoxic effects such as alteration of vimentin distribution (135) and hypoxia-induced degradation of keratin filaments (136) have the potential to impact the subcellular localization of mitochondria and glycolytic enzymes creating microenvironments of enriched concentrations of ATP, although this is currently unknown.

Partitioning of the cell *via* the cytoskeleton, which has already been demonstrated (137) and speculated to play a role in regulating metabolism (132), could create microenvironments of high ATP demand. Alternatively, this feature of the cytoskeleton could aid in biomolecular condensate formation. If cells possess the ability to rearrange these divides, it is a potential mechanism by which energy or energy-producing complexes can be distributed throughout the cell. However, there is a need for more research in this important field of cellular biology.

### Posttranslational modifications

Posttranslational modifications drive many intracellular processes and include phosphorylation, ubiquitination, hydroxylation, and SUMOylation. Hypoxia increases the activity of mitogen-activated protein kinases such as Jun-N-terminal kinase, extracellular signal-regulated protein kinase, and p38 kinase (138). With this increase in phosphorylation activity, several studies have demonstrated an accompanied increase in glycolysis (139, 140). A kinase belonging to the proviral integration site for Moloney murine leukemia virus (PIM) family, PIM2, is upregulated *via* NF-κB (141) which in turn results in the phosphorylation of PFKFB4 by PIM2 promoting anaerobic glycolysis (142).

On the other hand, protein SUMOylation is increased during periods of hypoxia *via* SUMO-1 (119, 143, 144, 145, 146). Evidence demonstrating the interaction of SUMO-1 with HIF-1α promoting its stabilization (145, 146), and enhancement of glycolysis as well as localization of glycolytic enzymes have been associated with SUMO-1 (119). However, the mechanism by which SUMO-1 influences glycolytic activity and the physiological relevance of this is still to be elucidated. mUbc9, a SUMO-conjugating enzyme which links SUMO-1 with various other proteins, can interact with GLUT1 and GLUT4 ultimately modulating glucose transport (147). Overexpression of mUbc9 causes an increase in GLUT4 protein expression, yet conversely reduces the total cellular content of the GLUT1 protein (147). GLUT1 is primarily utilized in basal glucose transport, whereas GLUT4 is predominantly used to accelerate glucose transport (148), potentially allowing for a SUMOylation-dependent acceleration in glucose transport and an upregulation in overall cell metabolism raising the intracellular concentration of ATP. Alongside this, SUMO-activating enzyme 1 SUMOylation modulates glycolytic metabolism *via* regulation of pyruvate kinase activity (149).

Hypermethylation of promoter regions of genes increases during periods of hypoxia *via* reduction in the activity of oxygen-dependent ten-eleven translocation enzymes, which catalyze DNA demethylation in normoxia (150). Methylation of various proteins, enzymes, and transporters associated with energy metabolism have been implicated in altering glucose transport and ATP synthesis and have been reviewed elsewhere extensively (151).

While the above modifications induced by hypoxia are pertinent to this review in the sense of energy metabolism, their role in altering distribution (if any) remains largely unknown. Other research on hypoxia and HIF relating to posttranslational modifications has been expertly discussed elsewhere (152, 153).

### Chaperone proteins

Heat shock proteins (HSP) are a highly conserved family of proteins that respond to various stress stimuli and are involved in many intracellular processes such as molecular chaperoning of proteins (154, 155), interactions with the actin cytoskeleton (156), and apoptosis (157). HSP expression is regulated through heat shock factor (HSF), a transcription factor that binds to the promoter region of HSP genes (158). Hypoxia upregulates HSP (159, 160, 161, 162, 163, 164) and HSF has been shown to have many conserved hypoxia response elements regions indicating that the hypoxic upregulation of HSP through HSF is HIF-1-dependent (159).

Within the HSP family, HSP70 and HSP90 are of particular interest due to their recent associations with glycolysis and oxidative phosphorylation. HSP90 overexpression enhances glucose consumption, lactate production (165) and drives a modest increase in ATP levels (166). Conversely, inhibition of HSP90 caused by binding with the molecular drug SU086, impairs glycolysis (167). Overexpression of HSP70 also increases glycolytic activity with upregulation of phosphofructokinase and lactate dehydrogenase activity being observed; however, this is met with a decrease in oxidative phosphorylation (168). Despite this converse relationship between both methods of ATP production, overall intracellular ATP concentrations were not significantly different between control cells and those overexpressing HSP70 (168). While the mechanisms by which the increases in glycolysis *via* HSPs described above have not yet been described, it presents an interesting hypothesis considering their involvement in molecular chaperoning.

### Biomolecular condensates, mitochondrial networks, and selective ATP use

Liquid-liquid phase separated organelle systems are biomolecular condensates in which proteins and nucleic acids can compartmentalize and reform temporally and spatially. This is an interesting mechanism due to the possibility of transporting mRNA strands to localized areas in need of ATP. With this, it could then be possible to synthesize proteins related to ATP production at the site of ATP demand, rather than transporting already synthesized proteins. Although this concept is intriguing, there is much to learn about the mechanisms behind LLPS organelle formation. DEAD-box (DDX) ATPases are global regulators of phase-separated condensates (169). While there has been a lack of data connecting DDX ATPases to LLPS organelle formation in stress conditions such as hypoxia, a recent study showed that DDX 1 binds to and protects stress response mRNAs (170). This indicates that DDX proteins play a role in mediating LLPS formation and are possibly involved in the formation of a glycolytic metabolon. Yet, there is no clearly defined role for DDX ATPases in response to hypoxic stress, and this remains an area of investigation.

Furthermore, it has been implicated that c-Myc-responsive long noncoding RNA has the capability to functionally contribute to the formation of a metabolon comprised of glycolytic enzymes which is accompanied with an increase in cell survival as well as ATP production (171). Therefore, RNA could act as the backbone of LLPS condensate formation and transportation of the translation machinery could prove cost-efficient for cells rather than transporting a higher quantity of individual enzymes.

Alongside temporal and spatial LLPS organelle formation, mitochondrial networks can differ between cell types and tissues and is reliant on the energetic demands of the cell. It is possible that this network plays some role in the distribution of intracellular ATP despite the effects of hypoxia. While the hypoxic effects on mitochondria are mostly inhibitory, it is still a site of ATP production. The mitochondrial network could potentially have a role in effectively distributing the intracellular ATP that is produced by the mitochondria throughout this period of metabolic stress.

**Figure 4:**
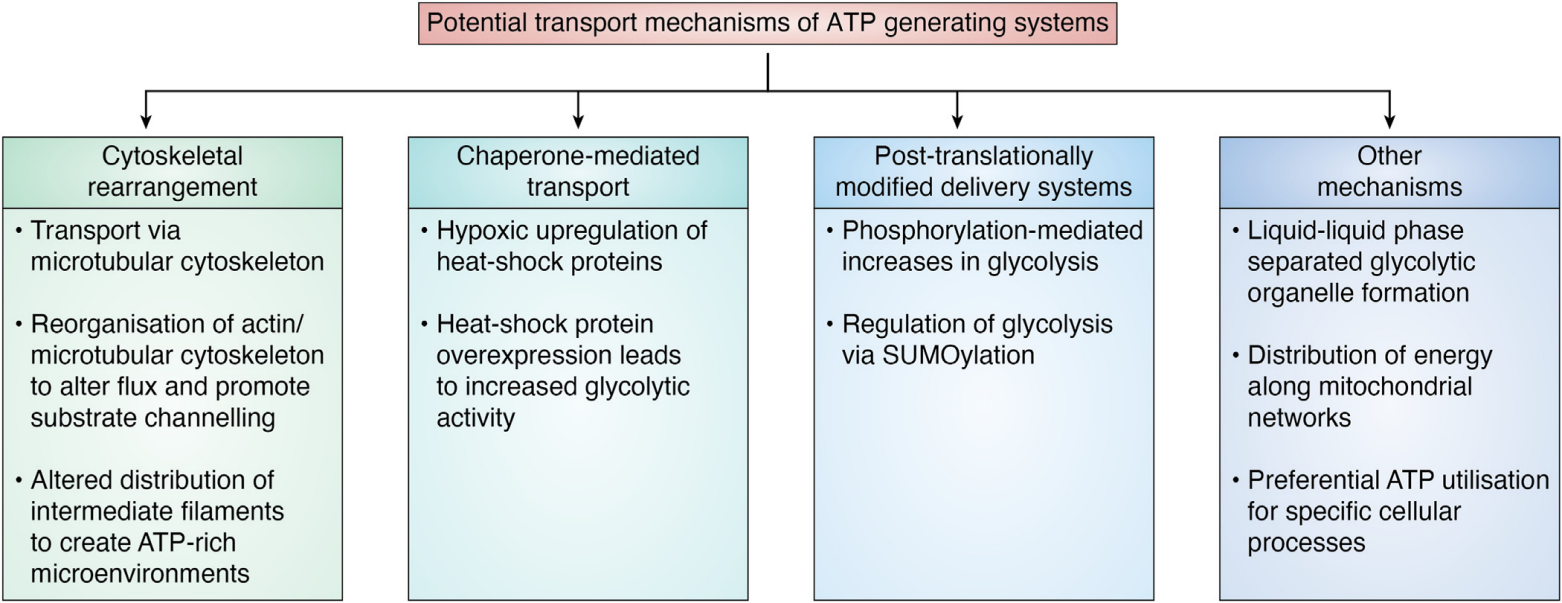
**Schematic represents a visual representation of possible mechanisms by which ATP-generating systems are directed throughout the cell.** This diagram highlights the key potential mechanisms by which a cell may distribute ATP in a targeted manner. These mechanisms include cytoskeletal rearrangement, chaperone-mediated transport, posttranslationally modified delivery systems, and others including liquid-liquid phase separated organelle formation, distribution along mitochondrial networks and preferential ATP utilization.

Interestingly, in differentiated podocytes inhibition of glycolysis reduced the formation of lamellipodia, decreased cell migration, and induced apoptosis of these cells whereas inhibition of the mitochondria had only minor effects on cell shape and migration (172). Alongside this, in prostate cancer, cells utilize ATP production from glycolysis for cytoskeletal remodeling and cell motility (173). The idea that cells can preferentially use methods of ATP production for specific cellular processes is intriguing considering the nonideal state of energy distribution where insufficient ATP is being produced by the cell. This begs the question: can processes that preferentially use mitochondrial ATP or glycolytic ATP resort to an alternative source of ATP or will the cell adapt to upregulate or increase the efficiency of the preferred method of ATP production? (Fig. 4).

However, methods in identification of these pathways have been insufficient. Emerging new strategies to monitor these ATP dynamics, which have been expertly discussed elsewhere recently (174, 175, 176, 177), could allow for the future identification and analysis of these mechanisms.

## Conclusion

In summary, the nonideal bioenergetic state induced by hypoxia presents the metazoan cell with a unique challenge in that ATP production becomes limited due to the decrease of available oxygen. While hypoxic adaptations are introduced, the reliance of the cell on the passive diffusion of ATP is likely to be insufficient to fuel important cellular processes. While this review highlights changes in energy production and potential mechanisms by which the mammalian cell may distribute ATP or ATP-producing organelles and complexes in hypoxia, there is still much research to be carried out before a full understanding of the intracellular distribution of ATP is uncovered.

## Conflict of interest

The authors declare that they have no conflicts of interest with the contents of this article.

## References

[1] Gordon J.L. (1986). Extracellular ATP: effects, sources and fate. Biochem. J..

[2] Dou L., Chen Y.-F., Cowan P.J., Chen X.-P. (2018). Extracellular ATP signaling and clinical relevance. Clin. Immunol..

[3] Khakh B.S., Burnstock G. (2009). The double life of ATP. Sci. Am..

[4] Wang Z., Cole P.A. (2014). Catalytic mechanisms and regulation of protein kinases. Methods Enzymol..

[5] Dunn J., Grider M.H. (2021). Physiology, adenosine triphosphate. StatPearls [Internet].

[6] Dhar-Chowdhury P., Malester B., Rajacic P., Coetzee W. (2007). The regulation of ion channels and transporters by glycolytically derived ATP. Cell. Mol. Life Sci..

[7] Katsuhara M., Mimura T., Tazawa M. (1990). ATP-regulated ion channels in the plasma membrane of a Characeae alga, Nitellopsis obtusa. Plant Physiol..

[8] Brokaw C.J. (1967). Adenosine triphosphate usage by flagella. Science.

[9] Daniel H.M., Fraden S., Nicastro D., Dogic Z. (2015). ATP consumption of eukaryotic flagella measured at a single-cell level. Biophys. J..

[10] Meurer F., Do H.T., Sadowski G., Held C. (2017). Standard Gibbs energy of metabolic reactions: II. glucose-6-phosphatase reaction and ATP hydrolysis. Biophys. Chem..

[11] Gordon D.M., Lyver E.R., Lesuisse E., Dancis A., Pain D. (2006). GTP in the mitochondrial matrix plays a crucial role in organellar iron homoeostasis. Biochem. J..

[12] ARAI T., KAZIRO Y. (1977). Role of GTP in the assembly of microtubules. J. Biochem..

[13] Rodbell M. (1992). The role of GTP-binding proteins in signal transduction: from the sublimely simple to the conceptually complex. Curr. Top. Cell. Regul..

[14] Sasaki A.T. (2016). Dynamic role of the GTP energy metabolism in cancers. Keio J. Med..

[15] Chang Y.-F., Carman G.M. (2008). CTP synthetase and its role in phospholipid synthesis in the yeast Saccharomyces cerevisiae. Prog. Lipid Res..

[16] Yitzhaki S., Hochhauser E., Porat E., Shainberg A. (2007). Uridine-5′-triphosphate (UTP) maintains cardiac mitochondrial function following chemical and hypoxic stress. J. Mol. Cell. Cardiol..

[17] Potter V.R. (1963). Feedback inhibition of thymidine kinase by thymidine triphosphate. Exp. Cell Res..

[18] Pinna S., Kunz C., Halpern A., Harrison S.A., Jordan S.F., Ward J. (2022). A prebiotic basis for ATP as the universal energy currency. PLoS Biol..

[19] Chu X.Y., Zhang H.Y. (2023). Prebiotic synthesis of ATP: a terrestrial volcanism-dependent pathway. Life.

[20] Akram M. (2014). Citric acid cycle and role of its intermediates in metabolism. Cell Biochem. Biophys..

[21] Lardy H.A., Ferguson S.M. (1969). Oxidative phosphorylation in mitochondria. Annu. Rev. Biochem..

[22] Hatefi Y. (1985). The mitochondrial electron transport and oxidative phosphorylation system. Annu. Rev. Biochem..

[23] Senior A.E. (1988). ATP synthesis by oxidative phosphorylation. Physiol. Rev..

[24] Slater E.C. (1977). Mechanism of oxidative phosphorylation. Annu. Rev. Biochem..

[25] Melkonian E.A., Schury M.P. (2022).

[26] Joost H.-G., Bell G.I., Best J.D., Birnbaum M.J., Charron M.J., Chen Y. (2002). Nomenclature of the GLUT/SLC2A family of sugar/polyol transport facilitators. Am. J. Physiol. Endocrinol. Metab..

[27] Robinson A.M., Williamson D.H. (1980). Physiological roles of ketone bodies as substrates and signals in mammalian tissues. Physiol. Rev..

[28] Schulz H. (1991). Beta oxidation of fatty acids. Biochim. Biophys. Acta.

[29] Greiner J.V., Glonek T. (2021). Intracellular ATP concentration and implication for cellular evolution. Biology (Basel).

[30] Heinrich R., Rapoport S.M., Rapoport T.A. (1978). Metabolic regulation and mathematical models. Prog. Biophys. Mol. Biol..

[31] Gan Q., Allen S.J., Taylor G. (2003). Kinetic dynamics in heterogeneous enzymatic hydrolysis of cellulose: an overview, an experimental study and mathematical modelling. Process Biochem..

[32] Meena A., Eswari A., Rajendran L., Serra P.A. (2011).

[33] Ovádi J., Tompa P., Vértessy B., Orosz F., Keleti T., Welch G.R. (1989). Transient-time analysis of substrate-channelling in interacting enzyme systems. Biochem. J..

[34] Kholodenko B.N., Rohwer J.M., Cascante M., Westerhoff H.V. (1998). Subtleties in control by metabolic channelling and enzyme organization. Mol. Cell. Biochem..

[35] Kholodenko B.N., Westerhoff H.V., Cascante C. (1996). Effect of channelling on the concentration of bulk-phase intermediates as cytosolic proteins become more concentrated. Biochem. J..

[36] Heerlein K., Schulze A., Hotz L., Bartsch P., Mairbaurl H. (2005). Hypoxia decreases cellular ATP demand and inhibits mitochondrial respiration of a549 cells. Am. J. Respir. Cell Mol. Biol..

[37] Semenza G.L. (1999). Regulation of mammalian O2 homeostasis by hypoxia-inducible factor 1. Annu. Rev. Cell Dev. Biol..

[38] Tian H., McKnight S.L., Russell D.W. (1997). Endothelial PAS domain protein 1 (EPAS1), a transcription factor selectively expressed in endothelial cells. Genes Dev..

[39] Gu Y.-Z., Moran S.M., Hogenesch J.B., Wartman L., Bradfield C.A. (1998). Molecular characterization and chromosomal localization of a third α-class hypoxia inducible factor subunit, HIF3α. Gene Expr. J. Liver Res..

[40] O'Rourke J.F., Tian Y.-M., Ratcliffe P.J., Pugh C.W. (1999). Oxygen-regulated and transactivating domains in endothelial PAS protein 1: comparison with hypoxia-inducible factor-1α. J. Biol. Chem..

[41] Wiesener M.S., Jürgensen J.S., Rosenberger C., Scholze C., Hörstrup J.H., Warnecke C. (2003). Widespread, hypoxia-inducible expression of HIF-2α in distinct cell populations of different organs. FASEB J..

[42] Jaakkola P., Mole D.R., Tian Y.-M., Wilson M.I., Gielbert J., Gaskell S.J. (2001). Targeting of HIF-α to the von Hippel-Lindau ubiquitylation complex by O_2_-regulated prolyl hydroxylation. Science.

[43] Knowles H.J., Raval R.R., Harris A.L., Ratcliffe P.J. (2003). Effect of ascorbate on the activity of hypoxia-inducible factor in cancer cells. Cancer Res..

[44] Pagé E.L., Chan D.A., Giaccia A.J., Levine M., Richard D.E. (2008). Hypoxia-inducible factor-1α stabilization in nonhypoxic conditions: role of oxidation and intracellular ascorbate depletion. Mol. Biol. Cell.

[45] Ivan M., Kondo K., Yang H., Kim W., Valiando J., Ohh M. (2001). HIFalpha targeted for VHL-mediated destruction by proline hydroxylation: implications for O2 sensing. Science.

[46] Yu F., White S.B., Zhao Q., Lee F.S. (2001). HIF-1α binding to VHL is regulated by stimulus-sensitive proline hydroxylation. Proc. Natl. Acad. Sci. U. S. A..

[47] Maxwell P.H., Wiesener M.S., Chang G.-W., Clifford S.C., Vaux E.C., Cockman M.E. (1999). The tumour suppressor protein VHL targets hypoxia-inducible factors for oxygen-dependent proteolysis. Nature.

[48] Cockman M.E., Masson N., Mole D.R., Jaakkola P., Chang G.-W., Clifford S.C. (2000). Hypoxia inducible factor-α binding and ubiquitylation by the von Hippel-Lindau tumor suppressor protein. J. Biol. Chem..

[49] Ohh M., Park C.W., Ivan M., Hoffman M.A., Kim T.-Y., Huang L.E. (2000). Ubiquitination of hypoxia-inducible factor requires direct binding to the β-domain of the von Hippel–Lindau protein. Nat. Cell Biol..

[50] Tanimoto K., Makino Y., Pereira T., Poellinger L. (2000). Mechanism of regulation of the hypoxia-inducible factor-1α by the von Hippel-Lindau tumor suppressor protein. EMBO J..

[51] McNeill L.A., Hewitson K.S., Claridge T.D., Seibel J.F., Horsfall L.E., Schofield C.J. (2002). Hypoxia-inducible factor asparaginyl hydroxylase (FIH-1) catalyses hydroxylation at the β-carbon of asparagine-803. Biochem. J..

[52] Lando D., Peet D.J., Gorman J.J., Whelan D.A., Whitelaw M.L., Bruick R.K. (2002). FIH-1 is an asparaginyl hydroxylase enzyme that regulates the transcriptional activity of hypoxia-inducible factor. Genes Dev..

[53] Kaelin W.G., Ratcliffe P.J. (2008). Oxygen sensing by metazoans: the central role of the HIF hydroxylase pathway. Mol. Cell.

[54] Jiang B.H., Semenza G.L., Bauer C., Marti H.H. (1996). Hypoxia-inducible factor 1 levels vary exponentially over a physiologically relevant range of O2 tension. Am. J. Physiol..

[55] Wood S.M., Gleadle J.M., Pugh C.W., Hankinson O., Ratcliffe P.J. (1996). The role of the aryl hydrocarbon receptor nuclear translocator (ARNT) in hypoxic induction of gene expression: studies in ARNT-deficient cells. J. Biol. Chem..

[56] Salceda S., Caro J. (1997). Hypoxia-inducible factor 1α (HIF-1α) protein is rapidly degraded by the ubiquitin-proteasome system under normoxic conditions: its stabilization by hypoxia depends on redox-induced changes. J. Biol. Chem..

[57] Huang L.E., Gu J., Schau M., Bunn H.F. (1998). Regulation of hypoxia-inducible factor 1α is mediated by an O2-dependent degradation domain via the ubiquitin-proteasome pathway. Proc. Natl. Acad. Sci. U. S. A..

[58] Kallio P.J., Wilson W.J., O'Brien S., Makino Y., Poellinger L. (1999). Regulation of the hypoxia-inducible transcription factor 1α by the ubiquitin-proteasome pathway. J. Biol. Chem..

[59] Huang L.E., Bunn H.F. (2003). Hypoxia-inducible factor and its biomedical relevance. J. Biol. Chem..

[60] Wang G.L., Jiang B.H., Rue E.A., Semenza G.L. (1995). Hypoxia-inducible factor 1 is a basic-helix-loop-helix-PAS heterodimer regulated by cellular O2 tension. Proc. Natl. Acad. Sci. U. S. A..

[61] Forsythe J.A., Jiang B.-H., Iyer N.V., Agani F., Leung S.W., Koos R.D. (1996). Activation of vascular endothelial growth factor gene transcription by hypoxia-inducible factor 1. Mol. Cell. Biol..

[62] Semenza G.L., Wang G.L. (1992). A nuclear factor induced by hypoxia via de novo protein synthesis binds to the human erythropoietin gene enhancer at a site required for transcriptional activation. Mol. Cell. Biol..

[63] Semenza G.L. (2011). Oxygen sensing, homeostasis, and disease. N. Engl. J. Med..

[64] Luo Z., Tian M., Yang G., Tan Q., Chen Y., Li G. (2022). Hypoxia signaling in human health and diseases: implications and prospects for therapeutics. Signal Transduct. Target. Ther..

[65] Koong A.C., Chen E.Y., Giaccia A.J. (1994). Hypoxia causes the activation of nuclear factor κB through the phosphorylation of IκBα on tyrosine residues. Cancer Res..

[66] Oliver K.M., Garvey J.F., Ng C.T., Veale D.J., Fearon U., Cummins E.P. (2009). Hypoxia activates NF-κB–dependent gene expression through the canonical signaling pathway. Antioxid. Redox Signal..

[67] Leeper-Woodford S.K., Detmer K. (1999). Acute hypoxia increases alveolar macrophage tumor necrosis factor activity and alters NF-κB expression. Am. J. Physiol..

[68] Matsui H., Ihara Y., Fujio Y., Kunisada K., Akira S., Kishimoto T. (1999). Induction of interleukin (IL)-6 by hypoxia is mediated by nuclear factor (NF)-κB and NF-IL6 in cardiac myocytes. Cardiovasc. Res..

[69] Schmedtje J.F., Ji Y.-S., Liu W.-L., DuBois R.N., Runge M.S. (1997). Hypoxia induces cyclooxygenase-2 via the NF-κB p65 transcription factor in human vascular endothelial cells. J. Biol. Chem..

[70] Beitner-Johnson D., Millhorn D.E. (1998). Hypoxia induces phosphorylation of the cyclic AMP response element-binding protein by a novel signaling mechanism. J. Biol. Chem..

[71] Leonard M.O., Howell K., Madden S.F., Costello C.M., Higgins D.G., Taylor C.T. (2008). Hypoxia selectively activates the CREB family of transcription factors in the in vivo lung. Am. J. Respir. Crit. Care Med..

[72] Salnikow K., Kluz T., Costa M., Piquemal D., Demidenko Z.N., Xie K. (2002). The regulation of hypoxic genes by calcium involves c-Jun/AP-1, which cooperates with hypoxia-inducible factor 1 in response to hypoxia. Mol. Cell. Biol..

[73] Damert A., Ikeda E., Risau W. (1997). Activator-protein-1 binding potentiates the hypoxia-induciblefactor-1-mediated hypoxia-induced transcriptional activation of vascular-endothelial growth factor expression in C6 glioma cells. Biochem. J..

[74] Millhorn D.E., Raymond R., Conforti L., Zhu W., Beitner-Johnson D., Filisko T. (1997). Regulation of gene expression for tyrosine hydroxylase in oxygen sensitive cells by hypoxia. Kidney Int..

[75] Cummins E.P., Taylor C.T. (2005). Hypoxia-responsive transcription factors. Pflügers Arch..

[76] Sermeus A., Michiels C. (2011). Reciprocal influence of the p53 and the hypoxic pathways. Cell Death Dis..

[77] Koizume S., Miyagi Y. (2015). Diverse mechanisms of Sp1-dependent transcriptional regulation potentially involved in the adaptive response of cancer cells to oxygen-deficient conditions. Cancers.

[78] Hardie D.G., Ashford M.L. (2014). AMPK: regulating energy balance at the cellular and whole body levels. Physiology.

[79] Hardie D.G., Schaffer B.E., Brunet A. (2016). AMPK: an energy-sensing pathway with multiple inputs and outputs. Trends Cell Biol..

[80] Hawley S.A., Davison M., Woods A., Davies S.P., Beri R.K., Carling D. (1996). Characterization of the AMP-activated protein kinase kinase from rat liver and identification of threonine 172 as the major site at which it phosphorylates AMP-activated protein kinase. J. Biol. Chem..

[81] Dengler F. (2020). Activation of AMPK under hypoxia: many roads leading to Rome. Int. J. Mol. Sci..

[82] Koumenis C., Naczki C., Koritzinsky M., Rastani S., Diehl A., Sonenberg N. (2002). Regulation of protein synthesis by hypoxia via activation of the endoplasmic reticulum kinase PERK and phosphorylation of the translation initiation factor eIF2α. Mol. Cell. Biol..

[83] Arsham A.M., Howell J.J., Simon M.C. (2003). A novel hypoxia-inducible factor-independent hypoxic response regulating mammalian target of rapamycin and its targets. J. Biol. Chem..

[84] Papandreou I., Cairns R.A., Fontana L., Lim A.L., Denko N.C. (2006). HIF-1 mediates adaptation to hypoxia by actively downregulating mitochondrial oxygen consumption. Cell Metab..

[85] Kim J.-W., Tchernyshyov I., Semenza G.L., Dang C.V. (2006). HIF-1-mediated expression of pyruvate dehydrogenase kinase: a metabolic switch required for cellular adaptation to hypoxia. Cell Metab..

[86] Zhang H., Gao P., Fukuda R., Kumar G., Krishnamachary B., Zeller K.I. (2007). HIF-1 inhibits mitochondrial biogenesis and cellular respiration in VHL-deficient renal cell carcinoma by repression of C-MYC activity. Cancer Cell.

[87] Li H.-S., Zhou Y.-N., Li L., Li S.-F., Long D., Chen X.-L. (2019). HIF-1α protects against oxidative stress by directly targeting mitochondria. Redox Biol..

[88] Zheng X., Narayanan S., Xu C., Angelstig S.E., Grünler J., Zhao A. (2022). Repression of hypoxia-inducible factor-1 contributes to increased mitochondrial reactive oxygen species production in diabetes. Elife.

[89] Fukuda R., Zhang H., Kim J.-W., Shimoda L., Dang C.V., Semenza G.L. (2007). HIF-1 regulates cytochrome oxidase subunits to optimize efficiency of respiration in hypoxic cells. Cell.

[90] Hori O., Ichinoda F., Tamatani T., Yamaguchi A., Sato N., Ozawa K. (2002). Transmission of cell stress from endoplasmic reticulum to mitochondria: enhanced expression of Lon protease. J. Cell Biol..

[91] Li Y., Lim S., Hoffman D., Aspenstrom P., Federoff H.J., Rempe D.A. (2009). HUMMR, a hypoxia-and HIF-1α–inducible protein, alters mitochondrial distribution and transport. J. Cell Biol..

[92] Ueda Y., Nakagawa T., Kubota T., Ido K., Sato K. (2005). Glioma cells under hypoxic conditions block the brain microvascular endothelial cell death induced by serum starvation. J. Neurochem..

[93] Yang L., Cao Z., Li F., Post D.E., Van Meir E.G., Zhong H. (2004). Tumor-specific gene expression using the survivin promoter is further increased by hypoxia. Gene Ther..

[94] Zhu L., Fukuda S., Cordis G., Das D.K., Maulik N. (2001). Anti-apoptotic protein survivin plays a significant role in tubular morphogenesis of human coronary arteriolar endothelial cells by hypoxic preconditioning. FEBS Lett..

[95] Bai H., Ge S., Lu J., Qian G., Xu R. (2013). Hypoxia inducible factor-1α-mediated activation of survivin in cervical cancer cells. J. Obstet. Gynaecol. Res..

[96] Rivadeneira D.B., Caino M.C., Seo J.H., Angelin A., Wallace D.C., Languino L.R. (2015). Survivin promotes oxidative phosphorylation, subcellular mitochondrial repositioning, and tumor cell invasion. Sci. Signal..

[97] Bleck C.K.E., Kim Y., Willingham T.B., Glancy B. (2018). Subcellular connectomic analyses of energy networks in striated muscle. Nat. Commun..

[98] Valente A.J., Fonseca J., Moradi F., Foran G., Necakov A., Stuart J.A., Urbani A., Babu M. (2019). Mitochondria in Health and in Sickness.

[99] Collins T.J., Berridge M.J., Lipp P., Bootman M.D. (2002). Mitochondria are morphologically and functionally heterogeneous within cells. EMBO J..

[100] Kuznetsov A.V., Schneeberger S., Renz O., Meusburger H., Saks V., Usson Y. (2004). Functional heterogeneity of mitochondria after cardiac cold ischemia and reperfusion revealed by confocal imaging. Transplantation.

[101] Kuznetsov A.V., Troppmair J., Sucher R., Hermann M., Saks V., Margreiter R. (2006). Mitochondrial subpopulations and heterogeneity revealed by confocal imaging: possible physiological role?. Biochim. Biophys. Acta.

[102] Glancy B. (2020). Visualizing mitochondrial form and function within the cell. Trends Mol. Med..

[103] Glancy B., Kim Y., Katti P., Willingham T.B. (2020). The functional impact of mitochondrial structure across subcellular scales. Front. Physiol..

[104] Liu X., Hajnoczky G. (2011). Altered fusion dynamics underlie unique morphological changes in mitochondria during hypoxia–reoxygenation stress. Cell Death Differ..

[105] Wu W., Lin C., Wu K., Jiang L., Wang X., Li W. (2016). FUNDC 1 regulates mitochondrial dynamics at the ER–mitochondrial contact site under hypoxic conditions. EMBO J..

[106] Ebert B.L., Firth J.D., Ratcliffe P.J. (1995). Hypoxia and mitochondrial inhibitors regulate expression of glucose transporter-1 via distinct cis-acting sequences. J. Biol. Chem..

[107] Vannucci S.J., Reinhart R., Maher F., Bondy C.A., Lee W.-H., Vannucci R.C. (1998). Alterations in GLUT1 and GLUT3 glucose transporter gene expression following unilateral hypoxia–ischemia in the immature rat brain. Brain Res. Dev. Brain Res..

[108] Semenza G.L., Roth P.H., Fang H.-M., Wang G.L. (1994). Transcriptional regulation of genes encoding glycolytic enzymes by hypoxia-inducible factor 1. J. Biol. Chem..

[109] Seagroves T.N., Ryan H.E., Lu H., Wouters B.G., Knapp M., Thibault P. (2001). Transcription factor HIF-1 is a necessary mediator of the pasteur effect in mammalian cells. Mol. Cell. Biol..

[110] Iyer N.V., Kotch L.E., Agani F., Leung S.W., Laughner E., Wenger R.H. (1998). Cellular and developmental control of O2 homeostasis by hypoxia-inducible factor 1α. Genes Dev..

[111] Giegé P., Heazlewood J.L., Roessner-Tunali U., Millar A.H., Fernie A.R., Leaver C.J. (2003). Enzymes of glycolysis are functionally associated with the mitochondrion in Arabidopsis cells. Plant Cell.

[112] Graham J.W., Williams T.C., Morgan M., Fernie A.R., Ratcliffe R.G., Sweetlove L.J. (2007). Glycolytic enzymes associate dynamically with mitochondria in response to respiratory demand and support substrate channeling. Plant Cell.

[113] Ginger M.L., McFadden G.I., Michels P.A. (2010). Rewiring and regulation of cross-compartmentalized metabolism in protists. Philos. Trans. R. Soc. B Biol. Sci..

[114] Araiza-Olivera D., Chiquete-Felix N., Rosas-Lemus M., Sampedro J.G., Peña A., Mujica A. (2013). A glycolytic metabolon in Saccharomyces cerevisiae is stabilized by F-actin. FEBS J..

[115] Sullivan D.T., MacIntyre R., Fuda N., Fiori J., Barrilla J., Ramizel L. (2003). Analysis of glycolytic enzyme co-localization in Drosophila flight muscle. J. Exp. Biol..

[116] Kohnhorst C.L., Kyoung M., Jeon M., Schmitt D.L., Kennedy E.L., Ramirez J. (2017). Identification of a multienzyme complex for glucose metabolism in living cells. J. Biol. Chem..

[117] Mamczur P., Dus D., Dzugaj A. (2007). Colocalization of aldolase and FBPase in cytoplasm and nucleus of cardiomyocytes. Cell Biol. Int..

[118] Puchulu-Campanella E., Chu H., Anstee D.J., Galan J.A., Tao W.A., Low P.S. (2013). Identification of the components of a glycolytic enzyme metabolon on the human red blood cell membrane. J. Biol. Chem..

[119] Agbor T.A., Cheong A., Comerford K.M., Scholz C.C., Bruning U., Clarke A. (2011). Small ubiquitin-related modifier (SUMO)-1 promotes glycolysis in hypoxia. J. Biol. Chem..

[120] Jin M., Fuller G.G., Han T., Yao Y., Alessi A.F., Freeberg M.A. (2017). Glycolytic enzymes coalesce in G bodies under hypoxic stress. Cell Rep..

[121] Fuller G.G., Han T., Freeberg M.A., Moresco J.J., Ghanbari Niaki A., Roach N.P. (2020). RNA promotes phase separation of glycolysis enzymes into yeast G bodies in hypoxia. Elife.

[122] Kierans S., Fagundes R.R., Malkov M., Sparkes R., Dillion E.T., Smolenski A. (2023). Hypoxia induces a glycolytic complex in intestial epithelial cells independent of HIF-1-driven glycolytic gene expression. Proc. Natl. Acad. Sci. U. S. A..

[123] Tong X., Tang R., Xu J., Wang W., Zhao Y., Yu X. (2022). Liquid–liquid phase separation in tumor biology. Signal Transduct. Target. Ther..

[124] Kierans S., Taylor C. (2021). Regulation of glycolysis by the hypoxia-inducible factor (HIF): implications for cellular physiology. J. Physiol..

[125] Kayyali U.S., Pennella C.M., Trujillo C., Villa O., Gaestel M., Hassoun P.M. (2002). Cytoskeletal changes in hypoxic pulmonary endothelial cells are dependent on MAPK-activated protein kinase MK2. J. Biol. Chem..

[126] Weidemann A., Breyer J., Rehm M., Eckardt K.-U., Daniel C., Cicha I. (2013). HIF-1α activation results in actin cytoskeleton reorganization and modulation of Rac-1 signaling in endothelial cells. Cell Commun. Signal..

[127] Yoon S.-O., Shin S., Mercurio A.M. (2005). Hypoxia stimulates carcinoma invasion by stabilizing microtubules and promoting the Rab11 trafficking of the α6β4 integrin. Cancer Res..

[128] Peng W.-X., Pan F.-Y., Liu X.-J., Ning S., Xu N., Meng F.-L. (2010). Hypoxia stabilizes microtubule networks and decreases tumor cell chemosensitivity to anticancer drugs through Egr-1. Anat. Rec. (Hoboken).

[129] Cao H., Yu D., Yan X., Wang B., Yu Z., Song Y. (2019). Hypoxia destroys the microstructure of microtubules and causes dysfunction of endothelial cells via the PI3K/Stathmin1 pathway. Cell Biosci..

[130] Hu J.-Y., Chu Z.-G., Han J., Dang Y-M, Yan H., Zhang Q. (2010). The p38/MAPK pathway regulates microtubule polymerization through phosphorylation of MAP4 and Op18 in hypoxic cells. Cell. Mol. Life Sci..

[131] Guo H., Zheng H., Wu J., Ma H.P., Yu J., Yiliyaer M. (2017). The key role of microtubules in hypoxia preconditioning-induced nuclear translocation of HIF-1α in rat cardiomyocytes. PeerJ.

[132] Cassimeris L., Silva V.C., Miller E., Ton Q., Molnar C., Fong J. (2012). Fueled by microtubules: does tubulin dimer/polymer partitioning regulate intracellular metabolism?. Cytoskeleton.

[133] Cooper G. (2000). Intermediate filaments. Cell Mol. Approach.

[134] Lowery J., Kuczmarski E.R., Herrmann H., Goldman R.D. (2015). Intermediate filaments play a pivotal role in regulating cell architecture and function. J. Biol. Chem..

[135] Liu T., Guevara O.E., Warburton R.R., Hill N.S., Gaestel M., Kayyali U.S. (2010). Regulation of vimentin intermediate filaments in endothelial cells by hypoxia. Am. J. Physiol.Cell Physiol..

[136] Na N., Chandel N.S., Litvan J., Ridge K.M. (2010). Mitochondrial reactive oxygen species are required for hypoxia-induced degradation of keratin intermediate filaments. FASEB J..

[137] Angert I., Karuka S.R., Mansky L.M., Mueller J.D. (2022). Partitioning of ribonucleoprotein complexes from the cellular actin cortex. Sci. Adv..

[138] Jin N., Hatton N., Swartz D.R., Xia X-L, Harrington M.A., Larsen S.H. (2000). Hypoxia activates Jun-N-terminal kinase, extracellular signal–regulated protein kinase, and p38 kinase in pulmonary arteries. Am. J. Respir. Cell Mol. Biol..

[139] Deng H., Yu F., Chen J., Zhao Y., Xiang J., Lin A. (2008). Phosphorylation of Bad at Thr-201 by JNK1 promotes glycolysis through activation of phosphofructokinase-1. J. Biol. Chem..

[140] Shu Y., Lu Y., Pang X., Zheng W., Huang Y., Li J. (2016). Phosphorylation of PPARγ at Ser84 promotes glycolysis and cell proliferation in hepatocellular carcinoma by targeting PFKFB4. Oncotarget.

[141] Blanco-Aparicio C., Carnero A. (2013). Pim kinases in cancer: diagnostic, prognostic and treatment opportunities. Biochem. Pharmacol..

[142] Lu C., Qiao P., Fu R., Wang Y., Lu J., Ling X. (2022). Phosphorylation of PFKFB4 by PIM2 promotes anaerobic glycolysis and cell proliferation in endometriosis. Cell Death Dis..

[143] Comerford K.M., Leonard M.O., Karhausen J., Carey R., Colgan S.P., Taylor C.T. (2003). Small ubiquitin-related modifier-1 modification mediates resolution of CREB-dependent responses to hypoxia. Proc. Natl. Acad. Sci. U. S. A..

[144] Nguyen H.-V., Chen J.-L., Zhong J., Kim K.-J., Crandall E.D., Borok Z. (2006). SUMOylation attenuates sensitivity toward hypoxia- or desferroxamine-induced injury by modulating adaptive responses in salivary epithelial cells. Am. J. Pathol..

[145] Shao R., Zhang F.-P., Tian F., Anders Friberg P., Wang X., Sjöland H. (2004). Increase of SUMO-1 expression in response to hypoxia: direct interaction with HIF-1α in adult mouse brain and heart in vivo. FEBS Lett..

[146] Bae S.-H., Jeong J.-W., Park J.A., Kim S.-H., Bae M.-K., Choi S.-J. (2004). Sumoylation increases HIF-1α stability and its transcriptional activity. Biochem. Biophys. Res. Commun..

[147] Giorgino F., de Robertis O., Laviola L., Montrone C., Perrini S., McCowen K.C. (2000). The sentrin-conjugating enzyme mUbc9 interacts with GLUT4 and GLUT1 glucose transporters and regulates transporter levels in skeletal muscle cells. Proc. Natl. Acad. Sci. U. S. A..

[148] Buse M.G., Robinson K.A., Marshall B.A., Mueckler M. (1996). Differential effects of GLUT1 or GLUT4 overexpression on hexosamine biosynthesis by muscles of transgenic mice. J. Biol. Chem..

[149] Wang C., Xiao Y., Lao M., Wang J., Xu S., Li R. (2020). Increased SUMO-activating enzyme SAE1/UBA2 promotes glycolysis and pathogenic behavior of rheumatoid fibroblast-like synoviocytes. JCI Insight.

[150] Thienpont B., Steinbacher J., Zhao H., D’Anna F., Kuchnio A., Ploumakis A. (2016). Tumour hypoxia causes DNA hypermethylation by reducing TET activity. Nature.

[151] Zhu X., Xuan Z., Chen J., Li Z., Zheng S., Song P. (2020). How DNA methylation affects the Warburg effect. Int. J. Biol. Sci..

[152] Kumar G.K., Klein J.B. (2004). Analysis of expression and posttranslational modification of proteins during hypoxia. J. Appl. Physiol..

[153] Daly L., Brownridge P.J., Sée V., Eyers C.E. (2020). Oxygen-dependent changes in HIF binding partners and post-translational modifications regulate stability and transcriptional activity. bioRxiv.

[154] Gething M.-J., Sambrook J. (1992). Protein folding in the cell. Nature.

[155] Hartl F.U. (1996). Molecular chaperones in cellular protein folding. Nature.

[156] Mounier N., Arrigo A.-P. (2002). Actin cytoskeleton and small heat shock proteins: how do they interact?. Cell Stress Chaperones.

[157] Parcellier A., Gurbuxani S., Schmitt E., Solary E., Garrido C. (2003). Heat shock proteins, cellular chaperones that modulate mitochondrial cell death pathways. Biochem. Biophys. Res. Commun..

[158] Sorger P.K., Pelham H.R. (1987). Purification and characterization of a heat-shock element binding protein from yeast. EMBO J..

[159] Baird N.A., Turnbull D.W., Johnson E.A. (2006). Induction of the heat shock pathway during hypoxia requires regulation of heat shock factor by hypoxia-inducible factor-1. J. Biol. Chem..

[160] Benjamin I.J., Kröger B., Williams R.S. (1990). Activation of the heat shock transcription factor by hypoxia in mammalian cells. Proc. Natl. Acad. Sci. U. S. A..

[161] Cao J., Yang L., Wang L., Zhao Q., Wu D., Li M. (2021). Heat shock protein 70 attenuates hypoxia-induced apoptosis of pulmonary microvascular endothelial cells isolated from neonatal rats. Mol. Med. Rep..

[162] Hammerer-Lercher A., Mair J., Bonatti J., Watzka S.B., Puschendorf B., Dirnhofer S. (2001). Hypoxia induces heat shock protein expression in human coronary artery bypass grafts. Cardiovasc. Res..

[163] Wen Y., Hu J., Wang J., Liu X., Li S., Luo Y. (2021). Effect of glycolysis and heat shock proteins on hypoxia adaptation of Tibetan sheep at different altitude. Gene.

[164] Lichtenauer M., Zimmermann M., Nickl S., Lauten A., Goebel B., Pistulli R. (2014). Transient hypoxia leads to increased serum levels of heat shock protein-27,-70 and caspase-cleaved cytokeratin 18. Clin. Lab..

[165] Xu Q., Tu J., Dou C., Zhang J., Yang L., Liu X. (2017). HSP90 promotes cell glycolysis, proliferation and inhibits apoptosis by regulating PKM2 abundance via Thr-328 phosphorylation in hepatocellular carcinoma. Mol. Cancer.

[166] Kang X., Chen J., Hou J-F (2022). HSP90 facilitates stemness and enhances glycolysis in glioma cells. BMC Neurol..

[167] Rice M.A., Kumar V., Tailor D., Garcia-Marques F.J., Hsu E.-C., Liu S. (2022). SU086, an inhibitor of HSP90, impairs glycolysis and represents a treatment strategy for advanced prostate cancer. Cell Rep. Med..

[168] Wang L., Schumann U., Liu Y., Prokopchuk O., Steinacker J.M. (2012). Heat shock protein 70 (Hsp70) inhibits oxidative phosphorylation and compensates ATP balance through enhanced glycolytic activity. J. Appl. Physiol..

[169] Hondele M., Sachdev R., Heinrich S., Wang J., Vallotton P., Fontoura B.M. (2019). DEAD-box ATPases are global regulators of phase-separated organelles. Nature.

[170] Li L., Garg M., Wang Y., Wang W., Godbout R. (2022). DEAD box 1 (DDX1) protein binds to and protects cytoplasmic stress response mRNAs in cells exposed to oxidative stress. J. Biol. Chem..

[171] Zhu Y., Jin L., Shi R., Li J., Wang Y., Zhang L. (2022). The long noncoding RNA glycoLINC assembles a lower glycolytic metabolon to promote glycolysis. Mol. Cell.

[172] Ozawa S., Ueda S., Imamura H., Mori K., Asanuma K., Yanagita M. (2015). Glycolysis, but not Mitochondria, responsible for intracellular ATP distribution in cortical area of podocytes. Sci. Rep..

[173] Shiraishi T., Verdone J.E., Huang J., Kahlert U.D., Hernandez J.R., Torga G. (2015). Glycolysis is the primary bioenergetic pathway for cell motility and cytoskeletal remodeling in human prostate and breast cancer cells. Oncotarget.

[174] White D., Yang Q. (2022). Genetically encoded ATP biosensors for direct monitoring of cellular ATP dynamics. Cells.

[175] Koberstein J.N., Stewart M.L., Smith C.B., Tarasov A.I., Ashcroft F.M., Stork P.J. (2022). Monitoring glycolytic dynamics in single cells using a fluorescent biosensor for fructose 1, 6-bisphosphate. Proc. Natl. Acad. Sci. U. S. A..

[176] Zhang Z., Chen W., Zhao Y., Yang Y. (2018). Spatiotemporal imaging of cellular energy metabolism with genetically-encoded fluorescent sensors in brain. Neurosci. Bull..

[177] Ley-Ngardigal S., Bertolin G. (2022). Approaches to monitor ATP levels in living cells: where do we stand?. FEBS J..

